# Genetic testing women with newly diagnosed breast cancer: What criteria are the most predictive of a positive test?

**DOI:** 10.1002/cam4.5515

**Published:** 2022-12-21

**Authors:** Kelly A. Metcalfe, Steven A. Narod, Andrea Eisen, Aletta Poll, Neda Zamani, David McCready, Tulin D. Cil, Frances C. Wright, Jordan Lerner‐Ellis, Jeanna McCuaig, Tracy Graham, Ping Sun, Mohammad R. Akbari

**Affiliations:** ^1^ Lawrence S. Bloomberg Faculty of Nursing, University of Toronto Toronto Canada; ^2^ Women's College Research Institute Toronto Canada; ^3^ Sunnybrook Health Sciences Centre Toronto Canada; ^4^ Institute of Medical Science, Faculty of Medicine, University of Toronto Toronto Canada; ^5^ Princess Margaret Cancer Centre, University Health Network Toronto Canada; ^6^ Mount Sinai Hospital Toronto Canada; ^7^ Dalla Lana School of Public Health, University of Toronto Toronto Canada

## Abstract

**Background:**

Knowledge of pathogenic variants in cancer‐predisposing genes is important when making breast cancer treatment decisions, but genetic testing is not universal and criteria must be met to qualify for genetic testing. The objective of this study was to evaluate the pathogenic variant yield for nine cancer predisposition genes by testing criteria, singly and in combination.

**Methods:**

Women diagnosed with breast cancer between June 2013 and May 2018 were recruited from four centers in Toronto, Canada. Participants completed a demographics and family history questionnaire and clinical characteristics were collected from medical charts. Genetic testing was done for *BRCA1*, *BRCA2*, *PALB2*, *ATM*, *CHEK2*, *BRIP1*, *RAD51D*, *RECQL*, and *TP53*. Pathogenic variant frequencies were calculated according to five criteria (age ≤ 50, triple‐negative breast cancer, family history, bilateral breast cancer, or Jewish ethnicity).

**Results:**

Of the 1006 women studied, 100 women (9.9%) were found to have a pathogenic variant in one of the nine genes tested. The highest prevalence of pathogenic variants was found in women with triple‐negative breast cancer (23%). Of the 100 pathogenic variants detected, 78 were detected in women diagnosed at age 50 or less. A total of 96% of the mutations were identified with three criteria (age of diagnosis, family history, and triple‐negative status).

**Conclusions:**

Genetic testing criteria for women with breast cancer should include women with triple‐negative breast cancer, regardless of age. All women aged 50 years or below at time of breast cancer diagnosis should be offered genetic testing.

## Graphical abstract

All genetic testing criteria evaluated resulted in at least a 10% pathogenic variant positive rate, with the highest being in women with triple negative breast cancer (23.2%). For women who only met one of the identified criteria, positivity rates ranged between 2.0% (Jewish ethnicity) to 14.3% (synchronous bilateral breast cancer).

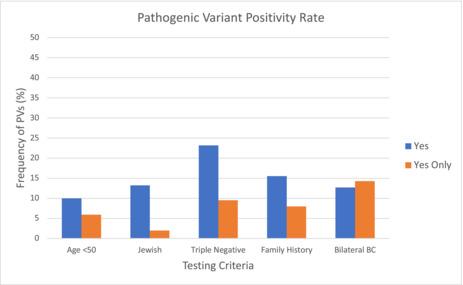

## 1 INTRODUCTION

Approximately, 10% of women with breast cancer carry a pathogenic variant in a cancer‐predisposing gene, including *BRCA1* and *BRCA2*.^1^ Knowledge of genetic status is becoming increasingly important for making breast cancer surgical and adjuvant treatment decisions. For women with breast cancer and a BRCA1 or BRCA2 pathogenic variant, bilateral mastectomy and oophorectomy have shown to improve survival.^2^, ^3^, ^4^ Recently it has been reported that for women with a *BRCA1* or *BRCA2* pathogenic variant, adjuvant Olaparib after local treatment and chemotherapy improves disease‐free survival.^5^ The American Society of Breast Surgeons recommends that genetic testing should be offered to all women with breast cancer,^6^ but this policy has not been universally adopted.

The cost of genetic testing has declined in recent years, and it has evolved from initially testing for *BRCA1* and *BRCA2* alone, to testing dozens of genes simultaneously. Panel testing expands our ability to identify women with a pathogenic variant; there are still criteria in place for who is eligible for testing to ensure that the women at highest risk are tested. Typically, these criteria were based on early studies of *BRCA1* and *BRCA2* and are based on age at breast cancer diagnosis, ethnicity (e.g., Jewish), clinical characteristics of breast cancer (e.g., bilateral breast cancer, triple‐negative breast cancer), and family history of cancer. However, the current eligibility criteria cannot capture all women with a pathogenic variant in the expanded gene panels, and women with a pathogenic variant are being missed.^7^, ^8^ Also, current criteria lists can be quite complex and challenging to the frontline care provider.

In 2013, we initiated a study of rapid genetic testing for women at the time of breast cancer diagnosis who were living in the Metropolitan Toronto region.^9^ Eligible women were newly diagnosed with breast cancer and satisfied one or more (of five) criteria. The current analysis is designed to evaluate these criteria, alone or in combination, on pathogenic variant yield. Yield can be expressed both in terms of the percentage of women in a given group who test positive or the total number patients in that group with a pathogenic variant. We can also estimate the incremental yield of adding each criterion step‐wise to the list. This information may help simplify genetic testing for non‐genetic professionals, including family doctors and breast surgeons, by identifying those criteria which are most informative.

## 2 METHODS

### 2.1 Study population

Ethical approval was obtained from all participating sites, which included four academic sites in Toronto, Canada (Women's College Hospital, Sunnybrook Health Sciences Centre, St. Michael's Hospital, and University Health Network). All women diagnosed with invasive breast cancer between June 2013 and May 2018 were identified. Women were eligible if this was the first primary invasive breast cancer, they were 18 years of age or older, had no previous prophylactic breast surgery or previous testing for *BRCA1* or *BRCA2* pathogenic variants and were able to understand and read English.

In addition, women had to meet at least one of the following criteria: Jewish ethnicity, triple‐negative breast cancer, age of diagnosis 50 or below, synchronous bilateral breast cancer, or a family history of breast cancer. A positive family history was defined as a first‐ or second‐degree relative with breast cancer diagnosed at age 50 or below or ovarian cancer or male breast cancer at any age.

### 2.2 Study procedures

Potentially eligible participants were identified by the treating surgeon at the time of breast cancer diagnosis. The surgeon referred the woman into the study, and within 24 hours, the woman was contacted by the genetic counselor, where eligibility was confirmed. Each participant provided written consent and were then provided with standard pre‐test genetic counseling and genetic testing was offered. The genetic counselor collected detailed information on family history and ethnicity to generate a three‐generation pedigree. Medical records were requested from the treating center, and relevant data (including pathology details) were abstracted by the genetic counselor.

### 2.3 Genetic testing

Participants provided a blood sample and genetic testing for *BRCA1* and *BRCA2* genes were performed using Sanger sequencing and MLPA (Multiplex Ligation Probe Amplification) assay. The archived DNA for women who consented to further testing was later tested for a panel of an additional seven genes including *ATM*, *CHEK2*, *BRIP1*, *PALB2*, *RAD51D*, *RECQL*, and *TP53* using the next‐generation sequencing technology. All genetic testing was done in our research laboratory and we used ACMG 5‐tier approach (Benign, Likely Benign, VUS, Likely Pathogenic and Pathogenic) for variant classification.

### 2.4 Statistical analysis

The analysis was descriptive. Frequencies of pathogenic variants by eligibility criteria were calculated for the study sample. All statistical tests were done by statistical software SAS version 9.4 (TS1M3), SAS Institute, Inc.

## 3 RESULTS

A total of 1006 women met eligibility for inclusion in the study, of whom 386 (28%) met more than one criterion for testing. The most common criterion for testing was age ≤ 50 years at breast cancer diagnosis (*n* = 783), followed by a family history of breast/ovarian cancer (*n* = 283), triple‐negative breast cancer at any age (*n* = 138), Jewish ethnicity (*n* = 121), and bilateral breast cancer (*n* = 71). Of these 1006 women, 100 women (9.9%) were found to have a pathogenic or likely pathogenic variant in one of the nine breast cancer susceptibility genes tested. Seventy‐nine (7.9%) were found to have a variant of unknown significance and 828 women (82.3%) had a negative genetic test result. Of the 100 pathogenic variants identified, 33 were found in *BRCA1*, 27 in *BRCA2*, 12 in *ATM*, 7 in *CHEK2*, 6 in *PALB2*, 4 in *BRIP1*, 4 in *RAD51D*, 4 in *RECQL*, and 3 in *TP53*. Six hundred and twenty women (72%) satisfied only one criterion for testing, of whom 7.7% were found to have a pathogenic variant.

Of the 100 women with pathogenic variants, 78 women were age 50 years or younger and 44 had a positive family history (Table 1). Smaller numbers had triple‐negative breast cancer (*n* = 32), were Jewish (*n* = 16), or had bilateral breast cancer (*n* = 9). However, for each of the five criteria, the pathogenic variant yield was 10% or greater. The highest percentage yield was for triple‐ negative breast cancer patients (23.2% positive) followed by those with a family history (15.5%).

**TABLE 1:** Yield of pathogenic variants by criterion

Criterion	Size of group	Number of pathogenic variants	Proportion positive	Percentage positive of total
Age < 50	783	78	10.0%	78%
Triple negative	138	32	23.2%	32%
Family history	283	44	15.5%	44%
Bilateral	71	9	12.7%	9%
Jewish	121	16	13.2%	16%
Any	1006	100	10.0%	100%

If we consider each criterion one at a time, we would have identified 78% of the pathogenic variant carriers by considering only age 50 or younger alone (Table 2). If we restricted ourselves to three criteria (age, triple‐negative receptor status, and family history), we would have identified 96% of the 100 carriers. Adding synchronous bilateral breast cancer extended the yield by three carriers, and adding Jewish ethnicity added a single carrier. A similar analysis was conducted but limited to the 60 BRCA1 and BRCA2 pathogenic variants (Table 3).

**TABLE 2:** Incremental yield of adding new criterion

Groups		Number of women	Number (%) pathogenic variants	Cumulative pathogenic variants
All subjects	Age < 50 Age ≥ 50	783 223	78 (10.0) 22 (9.9)	78
223 with age ≥ 50	Triple negative Non‐triple negative	41 182	10 (24.4) 12 (6.6)	88
182 with age ≥ 50 Non‐triple negative	Had family Hx No family Hx	111 71	8 (7.2) 4 (5.6)	96
71 with age ≥ 50, Non‐triple negative No family Hx	Had bilateral BC No bilateral BC	22 49	3 (13.6) 1 (2.0)	99
49 with age ≥ 50, Non‐triple negative, No family Hx, No bilateral BC	Jewish Non‐Jewish	49 0	1 (2.0)	100

**TABLE 3:** Incremental yield of adding new criterion (BRCA1 and BRCA2 only)

Groups		Number of women	Number (%) pathogenic variants in BRCA1/BRCA2	Cumulative pathogenic variants
All subjects	Age < 50 Age ≥ 50	783 223	49 (6.3) 11 (4.9)	49
223 with age ≥ 50	Triple negative Non‐triple negative	41 182	7(17.1) 4(2.2)	56
182 with age ≥ 50 Non‐triple negative	Had family Hx No family Hx	111 71	3(2.7) 1(1.4)	59
71 with age ≥ 50, Non‐triple negative No family Hx	Had bilateral BC No bilateral BC	22 49	1 (4.6) 0	60
49 with age ≥ 50, Non‐triple negative, No family Hx, No bilateral BC	Jewish Non‐Jewish	49 0	0 0	60

### 3.1 Age 50 years or younger at breast cancer

Overall, 10.0% of the women aged 50 or younger at the time of breast cancer were found to have a pathogenic variant in one of the nine genes (Table 4). A total of 62.8% of these pathogenic variants were detected in *BRCA1* and *BRCA2*. Of the 268 women 40 years or younger, 34 (12.7%) had a pathogenic variant. Of the 196 women 41–45 years, 16 (8.5%) had a pathogenic variant. Of the 226 women 46–50 years, 14 (6.2%) had a pathogenic variant.

**TABLE 4:** Pathogenic variants identified by Genetic Testing Criteria

Age ≤50	Total (1006)	*BRCA1* (33)	*BRCA2* (27)	*PALB2* (6)	*ATM* (12)	*CHEK2* (7)	*BRIP1* (4)	*RAD51D* (4)	*RECQL* (4)	*TP53* (3)	Negative/VUS	Any pathogenic variant (100)
No	223	8	3	3	2	2	1	2	1	0	201	22
	22.6%	24.2%	11.1%	50	16.67	28.57	25	50	25	0	22.19	9.7%
Yes	783	25	24	3	10	5	3	2	3	3	705	78
	77.4	75.76	88.89	50	83.33	71.43	75	50	75	100	77.81	10.0%
Yes only	492	3	7	2	7	3	1	1	3	2	463	29
	48.9	9.1	25.9	33.3	58.3	42.8	25	25	75	66.67	51.1	5.9%

Jewish												
No	885	26	21	6	11	5	4	4	4	3	801	84
	87.97	78.79	77.78	100	91.67	71.43	100	100	100	100	88.41	9.5%
Yes	121	7	6	0	1	2	0	0	0	0	105	16
	12.03	21.21	22.22	0	8.33	28.57	0	0	0	0	11.59	13.2%
Yes only	49	0	0	0	0	1	0	0	0	0	48	1
	4.87	0	0	0	0	14.3	0	0	0	0	5.3(45.71)	2.0%

Triple negative												
No	868	11	21	5	12	7	4	1	4	3	800	68
	86.28	33.33	77.78	83.33	100	100	100	25	100	100	88.3	7.8%
Yes	138	22	6	1	0	0	0	3	0	0	106	32
	13.72	66.67	22.22	16.67	0	0	0	75	0	0	11.7	23.2%
Yes only	21	1	1	0	0	0	0	0	0	0	19	2
	2.02	3	3.7	0	0	0	0	0	0	0	2.1(17.92)	9.5%

FamilyHx												
No	723	18	16	3	7	4	1	1	4	2	667	56
	71.87	54.55	59.26	50	58.33	57.14	25	25	100	66.67	73.62	7.7%
Yes	283	15	11	3	5	3	3	3	0	1	239	44
	28.13	45.45	40.74	50	41.67	42.86	75	75	0	33.33	26.38	15.5%
Yes only	87	1	2	1	1	1	1	0	0	0	80	7
	8.65	3	7.4	16.7	8.3	14.3	25	0	0	0	8.83	8.0%

Bilateral BC												
No	935	29	26	5	11	7	4	4	3	2	844	91
	92.94	87.88	96.3	83.33	91.67	100	100	100	75	66.67	93.16	9.7%
Yes	71	4	1	1	1	0	0	0	1	1	62	9
	7.06	12.12	3.7	16.67	8.33	0	0	0	25	33.33	6.84	12.7%
Yes only	21	1	0	1	0	0	0	0	1	0	18	3
	2.09	3	0	16.7	0	0	0	0	25	0	1.99	14.3%

For the 492 women who met the age criterion alone, 29 (5.9%) had a pathogenic variant, of which 10 (34.5%) were in *BRCA1* and *BRCA2* (Table 4).

### 3.2 Jewish ethnicity with breast cancer at any age

Overall, 16 of the 121 women (13.2%) who reported Jewish ancestry were found to have a pathogenic variant in one of the nine genes (Table 4). Of these, 13 (81.2%) were Jewish founder pathogenic variants in *BRCA1* or *BRCA2*. There were no pathogenic variants found in non‐Jewish founder pathogenic variants in *BRCA1* or *BRCA2*. The other pathogenic variants were detected in *ATM* (1) and *CHEK2* (2). No pathogenic variants were found in *PALB2*, *BRIP1*, *RAD51D*, *RECQL*, or *TP53*.

Of the 49 women who met only the Jewish ethnicity criterion, one woman (2.0%) was found to have a pathogenic variant in *CHEK2* (Table 4).

### 3.3 Triple‐negative breast cancer at any age

Overall, 23.2% of the women with triple‐negative breast cancer diagnosed at any age were found to have a pathogenic variant in one of the nine genes (Table 4). This was the highest prevalence of any criterion. *BRCA1* pathogenic variants accounted for the majority (22 of the 32) pathogenic variants in triple‐negative cancers (69%). Other pathogenic variants were detected in *BRCA2* (6), *RAD51D* (3), and *PALB2* (1).

There was no association with age of onset and mutation prevalence among the triple‐negative patients. Of the 97 women with triple‐negative breast cancer diagnosed at age 50 or younger, 22 (22.7%) had a pathogenic variant. Of the 26 women with triple‐negative breast cancer aged 51–60 years, six (23.1%) had a pathogenic variant. Of the 17 women aged 61 or older with triple‐negative breast cancer, four (23.2%) had a pathogenic variant.

Twenty‐one women met the triple‐negative breast cancer criterion only, and two (9.5%) had a pathogenic variant, all of which were in *BRCA1* and *BRCA2* (Table 4).

### 3.4 Family history of breast cancer

Overall, 28.1% of the women had a family history of breast cancer or ovarian cancer or male breast cancer. Of these, 44 (15.5%) were found to have a pathogenic variant in one of the nine genes (Table 4). Pathogenic variants were detected in all the genes tested, with the exception of *RECQL*. A total of 59.1% of the pathogenic variants were detected in *BRCA1* and *BRCA2*.

Of the 87 women who met family history criterion only, seven (8.0%) were found to have a pathogenic variant with 42.9% of these pathogenic variants in *BRCA1* and *BRCA2* (Table 4).

### 3.5 Synchronous bilateral breast cancer

Overall, 7.0% of the women were diagnosed with synchronous bilateral breast cancer and of these, nine (12.7%) were found to have a pathogenic variant. For the 21 women who met synchronous bilateral breast cancer criterion only, three (14.3%) had a pathogenic variant (one in each *BRCA1*, *PALB2*, and *RECQL*) (Table 4).

## 4 DISCUSSION

In 2022, most women with breast cancer who qualify for genetic testing receive panel genetic testing, where multiple genes are interrogated simultaneously. Most countries, including Canada, have criteria that a woman must meet in order to be offered genetic testing. There has been concern that by relying on restrictive testing criteria, women with a pathogenic variant in a cancer‐predisposition gene are being missed.^10^ This has implications for breast cancer treatment (including surgery and systemic therapy), screening and prevention of other cancers, and cascade testing for at‐risk relatives. In this study, we report on the prevalence of pathogenic variants by individual genetic testing criteria in a large cohort of women with breast cancer. Most women met a single criterion for testing, and the overall prevalence of pathogenic variants in the nine breast cancer predisposition genes tested was 9.9%.

In this study, we offered testing women with triple‐negative breast cancer, regardless of age at diagnosis. The frequency of pathogenic variants in women with triple negative breast cancer was 23.2%, with 91% of the observed pathogenic variants detected in *BRCA1*, *BRCA2*, and *PALB2*. Interestingly, there were no differences in frequency of pathogenic variants by age at diagnosis. For those who met the triple‐negative criterion alone, 9.5% had a pathogenic variant.

In Ontario, Canada, women with triple‐negative breast cancer qualify for genetic testing if they are diagnosed with breast cancer at age 60 or younger. The most recent 2022 NCCN guidelines for genetic testing have now removed the triple‐negative breast cancer age criterion, and triple‐negative breast cancer at any age is the updated criterion.^11^ This revision to NCCN testing criteria comes in response to recent research that has questioned the age criteria for triple‐negative breast cancer. Recently, Boddicker et al. reported on the frequency of pathogenic variants in 806 population‐based women with triple‐negative breast cancer diagnosed over the age of 60 years.^12^ Pathogenic variants in high‐risk breast cancer predisposition genes (*BRCA1*, *BRCA2*, and *PALB2*) were found in 4.5% of older women with triple‐negative breast cancer. In a smaller study that included 130 women with triple‐negative breast cancer over the age of 60 years, 12.3% of the women were found to have a pathogenic variant in a breast cancer predisposition gene (*BRCA1*, *BRCA2*, *PALB2*, *ATM*, and *RAD51C*).^13^ Our findings add to the growing body of evidence that all women with triple‐negative breast cancer should be eligible for genetic testing, regardless of age at diagnosis.

Young age at breast cancer diagnosis has historically been a criterion for genetic testing. The NCCN criteria stipulates an age cut‐off of 50 years or below at the time of breast cancer diagnosis.^14^ However, in Canada, provincial genetic testing criteria based on age cut‐off ranges from age 30 to 45 years. In this study, we included women with breast cancer up to age 50 years, which expands the typical age criterion for testing in Canada. There were 226 women included in this study who were diagnosed between the ages of 46 and 50 years and normally would not have met the age criteria for testing. Of these women, pathogenic variants were detected in 14 (6.2%) of women.

This finding adds to the evidence that the age criterion of being 45 or younger at breast cancer diagnosis needs to be revisited.^15^, ^16^ Recently, Yadav et al. recommended that genetic testing criteria should be expanded to include all women with breast cancer diagnosed at age 65 years or younger. This recommendation was in response to their findings that the sensitivity of NCCN guidelines was 70% for nine predisposition genes and 87% for *BRCA1* and *BRCA2*. By expanding the NCCN criteria to include age 65 or younger, the sensitivity improved to >90% for nine predisposition genes and > 98% for *BRCA1* and *BRCA2*.

In this study, 78% of the women tested were aged 50 or below at the time of breast cancer diagnosis, and this was the most common criterion met. This age criteria also yielded the highest number of pathogenic variants. For the entire cohort, 100 pathogenic variants were found, and 78 (78%) of those were women who were age 50 or younger at breast cancer diagnosis. If the goal of genetic testing is to identify the most women with pathogenic variants in breast cancer predisposition genes, the age criteria of breast cancer diagnosis would yield the highest number of pathogenic variant carriers.

The NCCN criteria for genetic testing includes any woman with breast cancer of Ashkenazi Jewish descent, due to the established frequency of pathogenic variants in *BRCA1* and *BRCA2* in this population.^17^ In this study, we have reported that for all Jewish women tested, 13.2% were found to have a pathogenic variant in one of the genes tested. However, for women who only met Jewish ethnicity criterion, 2% were identified with a pathogenic variant (1 *CHEK2* pathogenic variant). This yield of 2% in Jewish women with breast cancer who did not meet any other criteria is lower than what has been reported for women with breast cancer who did not meet any NCCN criteria which is approximately 3.5%.^8^, ^15^

There are limitations to our study. This study did not include all unselected women with breast cancer. In particular, women with a prior cancer in the contralateral breast were excluded. As a result, the findings from this study cannot be generalized to the overall breast cancer population. Furthermore, the patients were referred into this study from academic centers in a large metropolitan city in Canada with universal health care, and may not reflect breast cancer patients in community settings and other countries. As a result, health insurance status was not a limiting factor to participating in this study, and may be more representative of the breast cancer population. Furthermore, we offered testing to the women based on one of the five criteria and cannot say how many pathogenic variants were missed among women who did not meet any of these criteria. In addition, the genes that were tested in this study are not inclusive of all genes in which there are now data to support breast cancer risk association, including BARD1, RAD51C, and PTEN. We also included RECQL and BRIP1, in which there is conflicting evidence regarding the contribution to breast cancer.^18^, ^19^, ^20^, ^21^, ^22^, ^23^, ^24^ This may have led to an over‐ or under‐reporting of prevalence of pathogenic variants in this cohort.

Genetic testing criteria was established to ensure that women with the highest risk of having a pathogenic variant in a cancer predisposition gene are identified. In this study, 78% of the pathogenic variants identified were in women aged 50 years or younger at the time of breast cancer diagnosis. If the goal is to identify the highest yield of pathogenic variants, age 50 years or younger at time of breast cancer diagnosis is the single criteria that would identify the greatest number of pathogenic variant carriers. Genetic testing criteria may need to be expanded in various jurisdictions, to ensure that women with genetic pathogenic variants are being identified and can benefit from targeted treatments with superior survival outcomes.

## AUTHOR CONTRIBUTIONS

**Kelly A. Metcalfe:** Conceptualization (equal); data curation (equal); writing – original draft (equal); writing – review and editing (equal). **Steven Narod:** Data curation (equal); writing – original draft (equal); writing – review and editing (equal). **Andrea Eisen:** Data curation (equal); writing – review and editing (equal). **Aletta Poll:** Data curation (equal); writing – review and editing (equal). **Neda Zamani:** Data curation (equal); writing – review and editing (equal). **David McCready:** Data curation (equal); writing – review and editing (equal). **Tulin Cil:** Data curation (equal); writing – review and editing (equal). **Frances Catriona Wright:** Data curation (equal); writing – review and editing (equal). **Jordan Lerner‐Ellis:** Data curation (equal); writing – review and editing (equal). **Jeanna M McCuaig:** Data curation (equal); writing – review and editing (equal). **Tracy Graham:** Data curation (equal); writing – review and editing (equal). **Ping Sun:** Formal analysis (lead). **Mohammad R. Akbari:** Conceptualization (equal); data curation (equal); writing – review and editing (equal).

## FUNDING INFORMATION

This study was funded by a CIHR grant awarded to KM.

## CONFLICT OF INTEREST

The authors declare that there is no conflict of interest. MRA has ownership interest in Genewise Inc.

## Data Availability

The data that support the findings of this study are available on request from the corresponding author. The data are not publicly available due to privacy or ethical restrictions.
